# The prevalence of frailty and its relationship with sociodemographic factors, regional healthcare disparities, and healthcare utilization in the aging population across India

**DOI:** 10.1002/agm2.12263

**Published:** 2023-08-07

**Authors:** Sunny Singhal, Sumitabh Singh, Gevesh Chand Dewangan, Sharmistha Dey, Joyita Banerjee, Jinkook Lee, Ashish Datt Upadhyaya, Peifeng Hu, Aparajit Ballav Dey

**Affiliations:** ^1^ Department of Geriatric Medicine Sawai Man Singh Medical College and Hospital Jaipur India; ^2^ Department of Geriatric Medicine All India Institute of Medical Sciences Delhi India; ^3^ Department of Internal Medicine UT Southwestern Medical Center Dallas Texas USA; ^4^ Department of General Medicine All India Institute of Medical Sciences Raipur India; ^5^ Department of Biophysics All India Institute of Medical Sciences Delhi India; ^6^ Center for Economic and Social Research University of Southern California Los Angeles California USA; ^7^ Department of Biostatistics All India Institute of Medical Science Delhi India; ^8^ Division of Geriatric Medicine University of California, Los Angeles Los Angeles California USA; ^9^ Venu Geriatric Care Centre Delhi India

**Keywords:** low‐middle income country, social class, South‐Asia

## Abstract

**Objective:**

To estimate frailty prevalence and its relationship with the socio‐economic and regional factors and health care outcomes.

**Methods:**

In this study, participants from the harmonized Diagnostic Assessment of Dementia for the Longitudinal Aging Study in India (LASI‐DAD) were included. The frailty index (FI) was calculated using a 32‐variable deficit model, with a value of ≥ 25% considered as frail. Data on demographic (including caste and religion) and socioeconomic profiles and health care utilization were obtained. The state‐wise health index maintained by the government based on various health‐related parameters was used to group the participants' residential states into high‐, intermediate‐, and low‐performing states. Multivariable and zero‐inflated negative binomial regression was used to assess the relationship of frailty index with sociodemographic characteristics, health index, and health care expenditure or hospitalization.

**Results:**

Among the 3953 eligible participants, the prevalence of frailty was 42.34% (men = 34.99% and women = 49.35%). Compared to high‐performing states, intermediate‐ and low‐performing states had a higher proportion of frail individuals (49.7% *vs*. 46.8% *vs*. 34.5%, *P* < 0.001). In the adjusted analysis, frailty was positively associated with age, female sex, rural locality, lower education level, and caste (scheduled caste and other backward classes). After adjusting for the socio‐economic profile, FI was inversely associated with the composite health index of a state (*P* < 0.001). FI was also significantly correlated with total 1‐year health care expenditure and hospitalization (*P* < 0.001 and 0.020, respectively).

**Conclusion:**

There is a high prevalence of frailty among older Indian adults that is associated with sociodemographic factors and regional health care performance. Furthermore, frailty is associated with increased health care utilization and expenditure.

## Graphical abstract

Using nationally representative weighted sampling, the prevalence of frailty in the Indian population aged ≥ 60 years was found to be 42.34%. Frailty was positively associated with older age, female sex, low income, less education, living in a state with poor health index or rural areas, and belonging to the scheduled caste or other backward class (marginalized community). We also found that older adults with a higher degree of frailty experienced higher health expenditures and hospitalization rates in the previous year.

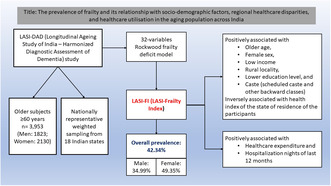

## 1 INTRODUCTION

Population aging, that is, increase in the number and proportion of older adults has shifted the focus of public health policies toward older adults.^1^ Frailty, a multifactorial clinical syndrome characterized by a decrease in the homeostatic or physiological reserve, is associated with increased vulnerability to adverse health outcomes,^2^ such as falls,^3^ disability,^4^ institutionalization,^5^ and death.^6^ It is a multidimensional syndrome caused by deficits in physical, psychological, and/or social domains.^7^ It is also a better predictor of biological age than chronological age,^8^ and measuring its prevalence in the community can play a key role in identifying the true burden of aging. Along with physiological health, frailty has also been linked to social determinants of health, and people who are socially disadvantaged are known to face a higher burden of frailty.^9^

India, the country with the second largest geriatric population globally,^10^ has a unique and complex social structure. Cowling et al^11^ reported differences in the social determinants of health across different states, castes, sexes, and urbanicity in the Indian population. This study states that the population belonging to underdeveloped states, those of the scheduled castes/scheduled tribes, those living in rural areas, and women face the highest inequities. This inequality was then translated into differences in life expectancy within different castes, religions, and regions.^12^ To improve population health and reduce regional disparities, a composite health index is calculated for each Indian state based on 23 indicators grouped into domains of health outcomes (neonatal mortality rate, tuberculosis [TB], and HIV cases, etc.), governance and information (medical officer occupancy rate, etc.), and key inputs or processes (number of vacant health care providers, etc.). However, it lacks geriatric specific outcomes.^13^

South Asians and, in particular, Indians differ from other populations in terms of socioeconomic status, health care behavior, attitude, education status, and genotype.^14^, ^15^, ^16^ At the population level, there is vast heterogeneity within the Indian population as there are several regional, sociodemographic, and economic differences affecting health‐related characteristics of older people.^17^ However, very few studies have investigated the prevalence of frailty in Indian older adults, and these studies are limited due to their small sample sizes and designs, therefore lacking generalizability.^18^, ^19^ Hence, a national sample, representative on the population level, is required to accurately estimate the burden of frailty. Further investigation of the association between frailty and sociodemographic factors, health care availability, utilization, and financing will help us in administering a targeted approach when managing the geriatric population of this largely diverse country.

To bridge this critical knowledge gap, we designed the present study with the following aims: (1) to construct a frailty index and report its prevalence among older Indian population; (2) to determine the association between frailty status and determinants of socioeconomic inequalities (income, education status, urbanicity, caste, and religion) and regional health care performance; and (3) to determine the correlation between the frailty index and health care‐related outcomes (total health care expenditure and total number of nights spent in the hospital in the last 12 months).

## 2 MATERIALS AND METHODS

### 2.1 Settings and study design

The data were obtained from the harmonized Diagnostic Assessment of Dementia for the Longitudinal Aging Study in India (LASI‐DAD). The Longitudinal Aging Study in India (LASI) is an ongoing cohort study funded by the National Institute on Aging (R01AG042778) and the Government of India. It is a nationally representative survey of the Indian population aged 45 years and older. LASI‐DAD is a part of the LASI, which includes a subsample of selected LASI respondents aged 60 years and above and is designed to collect data on late‐life cognition. It includes a subsample of selected LASI respondents aged 60 years and above from 18 states and union territories of India, thus representing 89% of the Indian population. Data are collected through a comprehensive geriatric assessment, including detailed cognitive interviews with both the respondents and caregivers. The LASI‐DAD uses a two‐stage stratified random sampling with oversampling of those at a high risk of cognitive impairment. In the first stage, LASI participants were stratified based on their state of residence and risk of cognitive impairment. In the second stage, an equal number of participants were randomly drawn from the two groups (high‐risk and low‐risk cognitive impairment) such that the sample size from each state was proportional to that included in the parent LASI study. Sample weights were created to account for this sampling strategy and nonresponders. Post‐stratification weights were computed using a raking algorithm that aligned the sample distributions of key demographic variables (age, sex, literacy, and urbanicity) to their population benchmarks (taken from the 2011 Indian Census). These post‐stratification weights allowed the LASI‐DAD to represent the population aged 60 years and above at the national level, although the sample was drawn from only 18 of the 28 states. The complete protocol for the LASI‐DAD has been published elsewhere.^20^

### 2.2 Construction of the LASI‐frailty index

There are various approaches to measure frailty.^21^ We used the deficit accumulation model, which defines frailty as the accumulation of various deficits across different physiologic systems.^22^ Its questionnaire‐based structure makes it ideal for use in community surveys and does not require significant training of interviewers.

Searle et al^23^ previously defined a standard procedure for selecting deficits and creating a frailty index (FI). Based on their definition, the variables selected must be associated with adverse health outcomes and may include symptoms, signs, disabilities, activities of daily living (ADL), self‐rated health, and comorbidities. The prevalence of impairment should generally increase with chronological age; however, it should not saturate too early. The variables included should cover a range of physiological systems and should avoid overweighing an individual system. Hence, the variables were excluded if the respective health domains were better represented by another available variable. The values for the variables must be present in more than 95% of the sample. Furthermore, the prevalence of the deficit should be more than 1% but less than 80% in the complete sample. Finally, the final constructed LASI‐FI must include at least 30–40 variables.

### 2.3 Scoring of the LASI‐FI

Only those participants with available values for ≥ 90% of the LASI‐FI variables were included in the final analysis. Each variable was scored from 0 to 1, with 0 indicating the absence and 1 indicating the presence of a deficit. A score of 0.5 was given in some deficits for an intermediate response. An individual's FI was calculated as the sum of the scores of individual deficits divided by the total number of deficits with non‐missing values. For simplification, FI was multiplied by 100. As used in the Rockwood Frailty model, an individual was considered frail if the FI was ≥ 25 and prefrail if FI was 8–25.^24^

### 2.4 Covariates

Information on sociodemographic factors (age, sex, education, urbanicity, caste, and religion) and state of residence was obtained from the LASI‐DAD study, whereas information on the annual household income, total health care expenditure of the last 12 months, and the total number of nights spent being hospitalized in the last 12 months was obtained for the respective participants from the main LASI study.

### 2.5 Health index

As mentioned previously, the health index is an annual report card that measures the performance of the health care sector of various states. The health‐index report released in June 2019, analyzed the states' performance for the year 2017–2018.^13^ As the first wave of LASI‐DAD data collection was also done during the same period, this report card was selected for our study. The six states having the highest health index were grouped as high‐performing states, followed by six states that were intermediate‐performing states, and, last, six states that were low‐performing states.

### 2.6 Statistical analysis

Statistical analyses were performed using the STATA software (release 12.1, Stata Corp). Statistical significance was set at *P* < 0.05. We used the LASI‐DAD post‐stratification sampling weight to adjust for nonresponse and complex sampling design. The Chi‐squared test was used to compare categorical variables while Wilcoxon rank‐sum test was used to compare continuous variables with frail categories.

As the clustering effect (Intra‐class correlation = 0.09) on frailty due to state was negligible in LASI data, we used multivariable linear regression modeling to establish the relationship among the FI, composite health index, and other socioeconomic factors. Model 1 shows the unadjusted relationship between the FI and composite health index. In model 2, age and sex were added to the regression model. Household income was added to model 3, and locality and years of education were further added to model 4. Finally, model 5 included religion and caste.

Count regression analysis was used to evaluate the relationship of health care expenditure and number of hospitalized nights with FI. A zero‐inflated negative binomial regression (ZINB) was chosen for both due to over‐dispersion and evidence of excess zeros. ZINB is a mixture model in which the outcome distribution consists of two parts. The first part is a logistic model for predicting excessive zeroes (zero and not zero) and the second part is a negative binomial model to account for the over‐dispersed counts. Thus, ZINB provides two sets of coefficients and corresponding *P* values for models relating to the logistic and counts parts.^25^ Further, the performance of the ZINB model was tested against Negative Binomial (NB) model using the Akaike information criterion (AIC) and Bayesian information criterion (BIC).

## 3 RESULTS

A total of 32 variables were included in the final LASI‐FI (Table S1). Figure S1 shows the process for selecting variables for the LASI‐FI. Despite having missing data accounting for 7.8%, body mass index (BMI) was included in the final index, considering its significant role in predicting adverse outcomes in older adults.^26^ Of the 4096 subjects, 143 (3.5%) were excluded due to having missing values for more than 10% of the LASI‐FI variables. Thus, 3953 subjects were finally included in the study.

**FIGURE 1:**
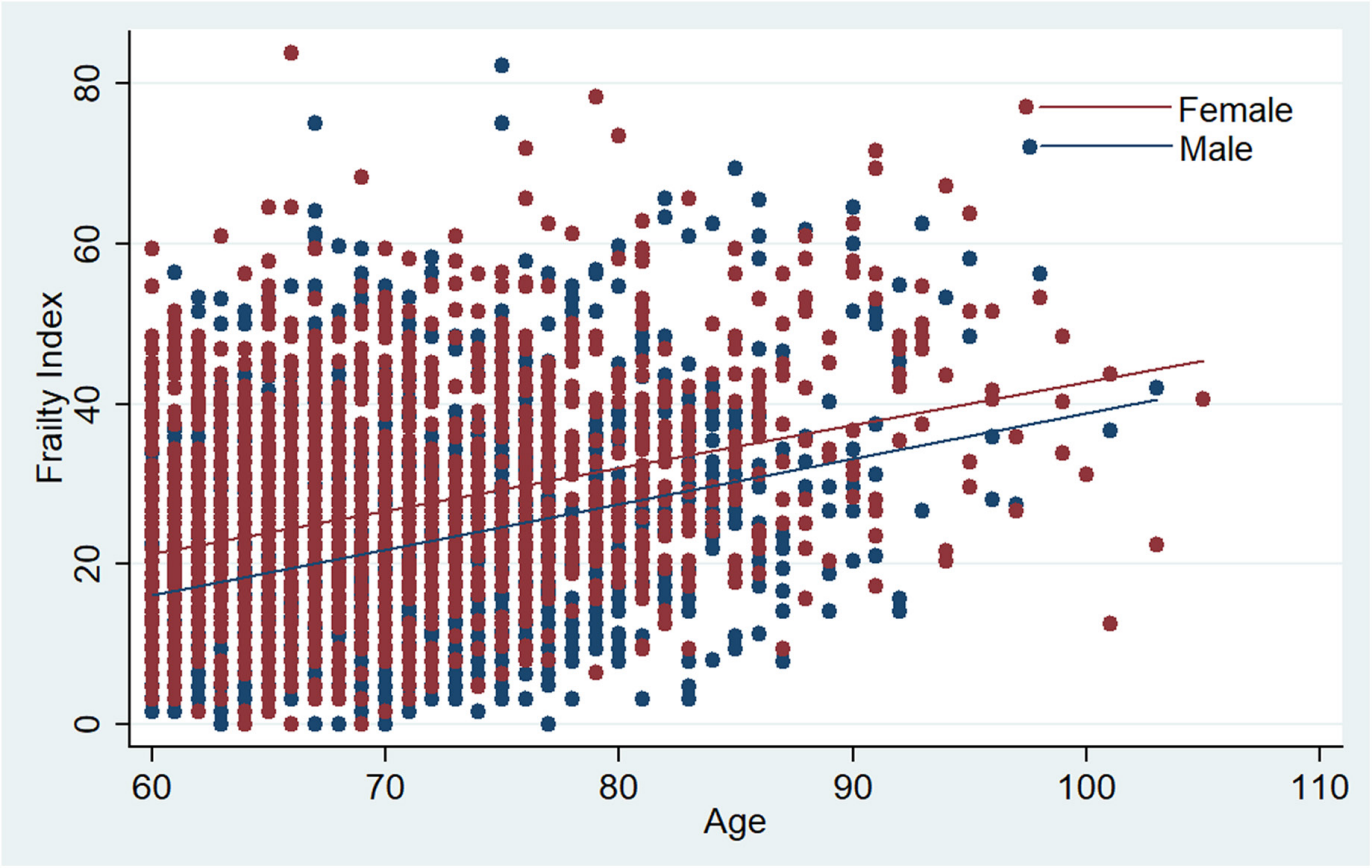
Scatter plot with fit‐line plot showing variation of frailty index with age in male participants (blue dots and line) and female participants (maroon dots and line).

Table 1 provides a description of the characteristics of the study population. The mean age of the population was 69.8 ± 0.2 years, and individuals with frailty were older compared to non‐frail individuals. The mean FI of the population was 23.5 ± 0.3 (women = 25.7 ± 0.4 and men = 21.2 ± 0.4). It increased with age and was higher in female patients (Figure 1). The prevalence of frailty, pre‐frailty, and non‐frailty were 42.34%, 47.64%, and 10.02%, respectively. In addition to older age and female sex, lower income, lower education, and rural locality were also associated with a high prevalence of frailty. As regard to caste, frailty was highest in the scheduled tribes (STs), followed by the scheduled castes (SCs), other backward classes (OBCs), and least in other or no caste. Similarly, regarding religion, the prevalence of frailty was highest among Hindus, followed by Muslims and other religions. Both health care expenditure and hospitalization duration were higher among individuals with frailty, but only differences in health care expenditure were statistically significant.

**TABLE 1:** Frailty status and baseline characteristics of the Diagnostic Assessment of Dementia for the Longitudinal Aging Study in India population.

	Total, n	Frail, n (proportion^a^)	Non‐frail, n (proportion^a^)	*P* value
Total	3953	1769 (42.3)	2184 (57.7)	
Age, y				
Mean ± SE^b^	69.8 ± 0.2	72.1 ± 0.3	68.0 ± 0.2	**< 0.001**
60–69 y	2245	817 (32.4)	1428 (67.6)	**< 0.001**
70–79 y	1242	615 (49.9)	627 (50.1)	
≥ 80 y	466	337 (71.0)	129 (29.0)	
Sex				
Female	2130	1089 (49.3)	1041 (50.7)	**< 0.001**
Male	1823	680 (35.0)	1143 (65.0)	
Annual household income (USD)				
Mean ± SE^b^	2832.4 ± 369.2	1741.1 ± 101.8	3635.6 ± 634.3	**< 0.001**
Education				
Years of education^b^	3.8 ± 0.1	2.2 ± 0.1	5.0 ± 0.2	**< 0.001**
Less than lower secondary	2964	1517 (49.6)	1447 (50.4)	**< 0.001**
Upper secondary and vocational training	826	221 (21.7)	605 (78.3)	
Tertiary	163	31 (16.0)	132 (84.0)	
Locality				
Urban	1499	523 (35.5)	976 (64.5)	**< 0.001**
Rural	2454	1246 (50.9)	1208 (49.1)	
Caste				
No caste or other caste	1335	507 (34.0)	828 (66.0)	**< 0.001**
Scheduled caste	720	366 (47.7)	354 (52.3)	
Scheduled tribe	202	106 (53.0)	96 (47.0)	
Other backward class	1677	782 (45.2)	895 (54.8)	
Religion				
Hindu	3132	1417 (42.8)	1715 (57.2)	0.521
Muslim	505	223 (41.3)	282 (58.7)	
Other	316	129 (38.5)	187 (61.5)	
Total expenditure on health care in last 12 mo (USD)				
Mean ± SE^b^	118.1 ± 18.0	153.9 ± 38.8	91.8 ± 12.9	**0.010**
Number of nights spent in the hospital in last 12 mo				
Mean ± SE^b^	0.66 ± 0.10	0.84 ± 0.20	0.53 ± 0.09	0.069

*Note*: All bold *P* values are < 0.05 denoting significant results.

Abbreviation: USD, United States dollars.

^a^ Weighted proportions.

^b^ Weighted means ± standard error (SE).

**FIGURE 2:**
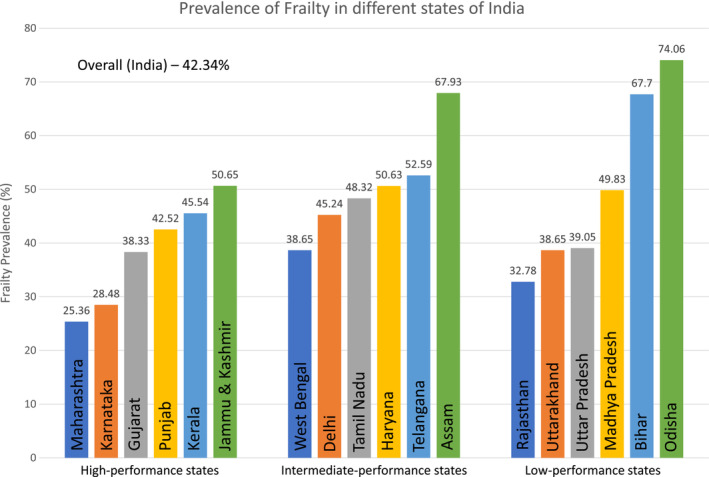
Prevalence of frailty in different states of India distributed as per the health index.

As shown in Figure 2, the highest prevalence was observed in Odisha (74.06%), whereas the lowest was observed in Maharashtra (25.36%). The prevalence of frailty and FI variables are presented in Table S2. The FI was higher in low‐performing states, followed by intermediate‐ and high‐performing states (Figure S2). The results from the multivariable linear regression analysis of the FI on health index and socioeconomic variables are shown in Table 2. From model 1 (unadjusted model), we see that the FI is inversely associated with the state's composite health, that is, frailty increases with poor performance of state on health index. After further adjustment for demographic and socioeconomic factors (age, sex, income, education, locality, caste, and religion) in models 2, 3, 4, and 5, the health index remained significantly associated with the FI. Similarly, older age, female sex, lower annual income, rural residence, and lower level of education were significantly associated with higher FI scores. Compared to no/other castes, belonging to SC or OBC was also associated with a higher FI.

**TABLE 2:** Multivariable linear regression analysis of the frailty index^a^, on the composite health index of the states and various socio‐economic variables.

	Model 1^b^	Model 2^b^	Model 3^b^	Model 4^b^	Model 5^b^
*R* ^2^	0.022	0.164	0.168	0.214	0.217
Composite Health Index	−0.147 (−0.190, −0.104)	−0.145 (−0.184, − 0.106)	−0.144 (−0.183, −0.104)	−0.086 (−0.127, −0.046)	−0.086 (−0.128, − 0.044)
Age, y		0.582 (0.513, 0.651)	0.578 (0.510, 0.647)	0.535 (0.468, 0.603)	0.539 (0.471, 0.607)
Sex (reference population – male) Female		4.992 (4.022, 5.963)	4.896 (3.929, 5.864)	3.333 (2.326, 4.329)	3.449 (2.445, 4.454)
Annual household income (per 1000 USD)			−0.059 (−0.085, −0.034)	−0.030 (−0.051, −0.009)	−0.032 (−0.051, −0.011)
Locality (reference population – urban) Rural				2.163 (1.131, 3.194)	2.044 (0.986, 3.103)
Years of education				−0.526 (−0.634, −0.417)	−0.490 (−0.603, −0.378)
Religion (reference population – Hindu) Muslim					0.254 (−1.286, 1.795)
Other					0.283 (−1.494, 2.060)
Caste (reference population – no caste or other caste) Scheduled caste					1.657 (0.223, 3.090)
Scheduled tribe					1.144 (−0.968, 3.256)
Other backward class					1.439 (0.322, 2.557)

*Note*: Model 1 was an unadjusted model; model 2 was adjusted for age and sex; model 3 was further adjusted for income; model 4 was further adjusted for locality and education; and model 5 was further adjusted for caste and religion.

^a^ The frailty index ranges from to 0–100.

^b^ The values reported are the regression coefficients (frailty index score) (95% confidence intervals) and are survey weighted.

**FIGURE 3:**
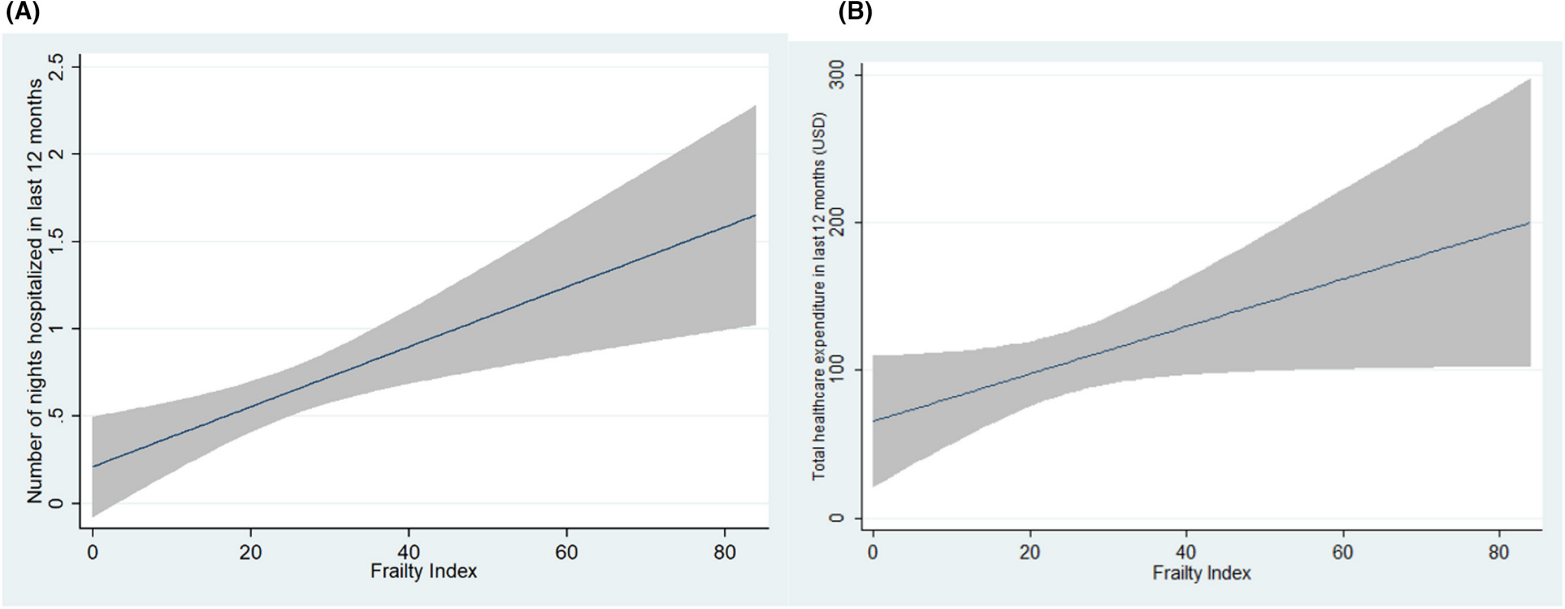
Fit‐line plot showing the relationship between frailty index and (A) number of nights spent in the hospital in last 1 year and (B) total health care expenditure in last 1 year.

Figure 3 shows the fit‐line plot between the frailty index and health care‐related outcomes. Table 3 shows the results of ZINB model of frailty index predicting number of nights spent in the hospital and health care expenditure in last 1 year. The FI was found to be significantly correlated with both total health care expenditure (*P* < 0.001) and total hospitalization duration (*P* = 0.020). Increased frailty was found to be associated with increased odds of both having hospitalization and/or health care expenditure in 1 year (logit model). Similarly, increased frailty was also associated with increased nights of hospitalization and amount of health care expenditure (count model). This correlation remained significant after adjusting for age and sex (adjusted *P* < 0.05 for both). As compared to NB model, the ZINB model had the lowest AIC (NB = 56988.38 and ZINB = 55619.96) and BIC coefficients (NB = 57007.23 and ZINB = 55651.36) for health care expenditure. Similarly, the ZINB model had the lowest AIC (NB = 4390.443 and ZINB = 4373.584) and BIC coefficients (NB = 4409.263 and ZINB = 4404.951) for hospitalization nights. Thus, indicating ZINB to be a better performing or more suitable model for both variables.

**TABLE 3:** Zero Inflated Negative Binomial (ZINB) Regression analysis results for health care expenditure and hospitalization nights in last 1 year.

	Total health care expenditure in last 1 y	Number of nights spent in the hospital in last 1 y
Logistic portion of model (yes/no)		
Coefficient	−0.006	−0.012
95% confidence interval	−0.011 to (−)0.001	−0.021 to (−)0.003
*P* value	**0.029**	**0.009**
Counts portion of model (amount/events)		
Coefficient	0.013	0.012
95% confidence interval	0.008 to 0.017	0.001 to 0.023
*P* value	**< 0.001**	**0.027**

*Note*: All bold *P* values are < 0.05 denoting significant results.

## 4 DISCUSSION

### 4.1 Prevalence of frailty

In our study, the weighted prevalence of frailty and pre‐frailty in Indian older adults was 42.34% and 47.64%, respectively. The prevalence of frailty was relatively high, which is comparable to that obtained from other Indian studies. The World Health Organization Study on Global Ageing and Adult Health (WHO‐SAGE study), which is the only other study with national sampling from six Indian states, reported a similar prevalence of frailty (44.5%).^27^ A few regional studies from eastern and southern India have reported a prevalence of frailty of as high as 59%.^28^, ^29^ Although one study reported a lower prevalence (26.1%), the study participants only belonged to an urban locality.^19^ Another study conducted in an outpatient setting also reported a similar prevalence of 44%.^18^ However, besides the WHO‐SAGE study, all other community‐based studies included participants from a single state only and hence lacked generalizability. Although the study population of the WHO‐SAGE included participants from all regions, it included only one state from one region. In our study, we included multiple states from one region to capture intra‐regional heterogeneity. Furthermore, the FI used in the WHO‐SAGE study lacks variables from the cognitive domain,^27^ which is a major component of geriatric health. By including variables assessing cognitive function, we aimed to create a more robust FI.

Although the prevalence of frailty varies with the study tool used, earlier studies have also shown that frailty is more prevalent in low‐middle‐income countries (such as India) than in high‐income countries.^30^, ^31^, ^32^ Socioeconomic inequalities (lower education and wealth)^33^, ^34^ and health care disparities (poor accessibility, quality, and increased cost)^35^, ^36^ may explain the increased prevalence of frailty in such countries. Ethnicity also plays an important role in frailty. An earlier study conducted in the United Kingdom showed a higher prevalence of frailty among South Asians (including Indians) compared to White and Black ethnic groups,^37^ which can be related to their high cardiovascular risk profile.^38^

### 4.2 Relationship of frailty with sociodemographic factors

In our study, there was an increase in the prevalence of frailty with age, as demonstrated by frailty being more prevalent in the oldest old (≥ 80 years). Frailty was more prevalent among women than men. A higher proportion of people who were frail also undertook fewer years of education; there was a decrease in the prevalence of frailty with an increase in the years of education. These findings are consistent with those of the WHO‐SAGE study, where the oldest old and female participants showed higher levels of frailty than younger old and male participants. In addition, the mean frailty score demonstrated a strong inverse relationship with education, with lower levels of education showing higher levels of frailty.^39^

Individuals living in rural areas were more likely to be frail than those residing in urban areas. The frailty gap among urban–rural inhabitants can be explained by differences in their wealth index, education, physical activity, community engagement.^40^ Caste also showed significant variability in being frail, with a higher proportion of frail individuals belonging to SC, followed by ST. The WHO‐SAGE data demonstrated similar results, although they determined frailty based on the phenotypic model.^41^ Further studies are required to understand the reason for this difference in the prevalence of frailty among various caste groups. Religion of participants was not found to be significantly associated with frailty. However, larger sample size of other religious minorities (Sikhs, Christians, etc.) may be required for better understanding.

### 4.3 Relationship of frailty with health index of states

On comparing the FI with the health index of the states, we found it to be inversely correlated, that is, states with a better health index were more likely to have a lower FI. On further classification of the states based on the health index, states with a better health index (high‐performing states) had a lower prevalence of frailty among older adults as compared to those with a lower health index (intermediate‐ and low‐performing states). This higher prevalence of frailty in low‐performing states could be due to a multitude of reasons, such as poor allocation and utilization of health care resources and improper implementation of health policies for older individuals. As frailty is strongly associated with mortality in older adults,^6^ the FI and health index can help identify states that have more vulnerable older adults, thus improving the focus on geriatric health care.

However, it is also important to note that the health outcomes used to develop the health index are primarily pediatric (neonatal, under‐5 mortality rate, etc.), maternal (institutional deliveries and total fertility rate), or infection‐related (TB and HIV) and do not have any chronic disease, disability, or other geriatric‐related outcome.^42^ This can explain the high prevalence of frailty in some high‐performing states and, similarly, the low prevalence in some low‐performance states (Figure 2). Furthermore, because of improvements in health and medical care, deaths from infections and maternal and perinatal causes are decreasing, whereas chronic non‐infectious diseases are becoming more common causes of morbidity and mortality.^43^ We need to develop better data systems that can measure such geriatric‐related outcomes (eg, FI) to understand the health risks faced by older people. These data systems can be used for health care policy and decision making to target appropriate prevention and intervention services and strengthen further research.

### 4.4 Health care outcomes and frailty index

As seen in the ZINB model (Table 3), the odds and amount of both health care‐related expenditures and duration of hospitalization increases with increased frailty. This finding was simulated by previous studies,^44^ particularly the ESTHER study,^45^ where frailty was found to be an important and significant factor for an increase in health care costs independent of age and comorbidity. Although it is a well‐known fact that frailty increases the risk of hospitalization among older adults,^46^, ^47^ our study further strengthens the fact that increasing frailty, as determined by an increase in the FI, leads to an increased length of hospital stay.

### 4.5 Strengths and limitations

The major strength of this study is its strong sampling strategy. As mentioned earlier, the LASI‐DAD is a nationally representative weighted survey that recruited subjects from 18 different states and represented 89% of the population, thereby increasing the generalizability and applicability of our study. As this was designed to be a longitudinal study, this index can also be used for future analysis. Furthermore, our study provides an understanding of how regional health care differences can be associated with frailty in older adults, which was previously unexplored. Last, sociodemographic factors, such as caste and religion, which play an important role in determining the health status of an individual,^48^ were also included in this study.

This study had a few limitations. First, because we used cross‐sectional data, causation could not be established. However, as data from subsequent waves become available, we will use this model for further validation. Second, we used the deficit accumulation model to measure frailty. However, it is known to overestimate prevalence as compared to the phenotypic model.^30^, ^31^ As other data (grip strength and gait speed) from the LASI‐DAD are being collected, we will, in the future, need to compare prevalence between the two models for better understanding.

## 5 CONCLUSION

India has a high prevalence of frailty among older individuals, and this is associated with various demographic and socio‐economic factors. Frailty is also inversely associated with the health care performance of a state. Furthermore, it is associated with increased hospitalization duration and health care expenditure. Using frailty as either a health care variable or outcome in a state's policymaking strategy can help improve the assessment and delivery of health services to older adults.

## AUTHOR CONTRIBUTIONS

S. Singhal and S. Singh conceptualized the study and its methodology. Data acquisition and project supervision was done by A. B. D., S. D., J. B., J. L., and P.H. S. Singhal, S. Singh, G. C. D., and A. D. U. performed the study analysis and wrote the original draft. All authors reviewed and gave approval for the final version.

## FUNDING INFORMATION

This work was supported by the National Institute on Aging R01 AG051125.

## CONFLICT OF INTEREST STATEMENT

The authors declare that they have no competing interests.

## ETHICS APPROVAL

This study is based on anonymous data obtained from the LASI (Longitudinal Aging Study of India) and the LASI‐DAD (Longitudinal Aging Study of India – Harmonized Diagnostic Assessment of Dementia). Both the original LASI and LASI‐DAD study were approved by the human ethics committee of the All India Institute of Medical Sciences (New Delhi) (IEC‐284/06.05.2016, RP‐33/2016), the Indian Council of Medical Research (New Delhi), and the International Institute of Population Sciences (Mumbai).

## CONSENT FOR PUBLICATION

Participants provided consent for the anonymous publication of data.

## Supporting information


Appendix S1.

## Data Availability

The original cohort data for LASI (https://www.iipsindia.ac.in/content/LASI‐data) and LASI‐DAD population (https://g2aging.org/downloads) is publicly available and can be accessed through the aforementioned weblinks.

## References

[1] Global Strategy and Action Plan on Ageing and Health. World Health Organisation; 2017. Accessed February 23, 2022. https://www.who.int/publications‐detail‐redirect/9789241513500

[2] Morley JE , Vellas B , Abellan van Kan G , et al. Frailty consensus: a call to action. J Am Med Dir Assoc. 2013;14(6):392‐397. doi:10.1016/j.jamda.2013.03.022 23764209 PMC4084863

[3] Zaslavsky O , Zelber‐Sagi S , Gray SL , et al. A comparison of frailty phenotypes for prediction of mortality, incident falls, and hip fractures in older women. J Am Geriatr Soc. 2016;64(9):1858‐1862. doi:10.1111/jgs.14233 27310179 PMC5026871

[4] Ensrud KE , Ewing SK , Cawthon PM , et al. A comparison of frailty indexes for the prediction of falls, disability, fractures and mortality in older men. J Am Geriatr Soc. 2009;57(3):492‐498. doi:10.1111/j.1532-5415.2009.02137.x 19245414 PMC2861353

[5] Kojima G . Frailty as a predictor of nursing home placement among community‐dwelling older adults: a systematic review and meta‐analysis. J Geriatr Phys Ther. 2018;41(1):42‐48. doi:10.1519/JPT.0000000000000097 27341327

[6] Shamliyan T , Talley KMC , Ramakrishnan R , Kane RL . Association of frailty with survival: a systematic literature review. Ageing Res Rev. 2013;12(2):719‐736. doi:10.1016/j.arr.2012.03.001 22426304

[7] Wleklik M , Uchmanowicz I , Jankowska EA , et al. Multidimensional approach to frailty. Front Psychol. 2020;11:564. doi:10.3389/fpsyg.2020.00564 32273868 PMC7115252

[8] Mitnitski A , Rockwood K . Aging as a process of deficit accumulation: its utility and origin. Interdiscip Top Gerontol. 2015;40:85‐98. doi:10.1159/000364933 25341515

[9] Dugravot A , Fayosse A , Dumurgier J , et al. Social inequalities in multimorbidity, frailty, disability, and transitions to mortality: a 24‐year follow‐up of the Whitehall II cohort study. Lancet Public Health. 2020;5(1):e42‐e50. doi:10.1016/S2468-2667(19)30226-9 31837974 PMC7098476

[10] United Nations, Department of Economic and Social Affairs . Population division. World Population Ageing, 2019 Highlights; 2020.

[11] Cowling K , Dandona R , Dandona L . Social determinants of health in India: progress and inequities across states. Int J Equity Health. 2014;13:88. doi:10.1186/s12939-014-0088-0 25294304 PMC4201685

[12] Kumari M , Mohanty SK . Caste, religion and regional differentials in life expectancy at birth in India: cross‐sectional estimates from recent National Family Health Survey. BMJ Open. 2020;10(8):e035392. doi:10.1136/bmjopen-2019-035392 PMC7440832 32819936

[13] Health Index. Accessed October 28, 2021. http://social.niti.gov.in/health‐index

[14] Bhopal R , Hayes L , White M , et al. Ethnic and socio‐economic inequalities in coronary heart disease, diabetes and risk factors in Europeans and south Asians. J Public Health Med. 2002;24(2):95‐105. doi:10.1093/pubmed/24.2.95 12141592

[15] Ayub Q , Tyler‐Smith C . Genetic variation in South Asia: assessing the influences of geography, language and ethnicity for understanding history and disease risk. Brief Funct Genomics. 2009;8(5):395‐404. doi:10.1093/bfgp/elp015 19535507

[16] Wechkunanukul K , Grantham H , Damarell R , Clark RA . The association between ethnicity and delay in seeking medical care for chest pain: a systematic review. JBI Database System Rev Implement Rep. 2016;14(7):208‐235. doi:10.11124/JBISRIR-2016-003012 27532797

[17] Dey S , Nambiar D , Lakshmi JK , Sheikh K , Reddy KS . Health of the Elderly in India: Challenges of Access and Affordability. National Academies Press; 2012. Accessed July 15, 2020. https://www.ncbi.nlm.nih.gov/books/NBK109208/

[18] Singhal S , Dewangan GC , Bansal R , et al. Sarcopenia and its association with geriatric syndromes and quality of life in older Indian outpatients–a cross‐sectional pilot observational study. J Indian Acad Geriatr. 2019;15(2):66‐74. doi:10.35262/jiag.v15i2.66-74

[19] At J , Bryce R , Prina M , et al. Frailty and the prediction of dependence and mortality in low‐ and middle‐income countries: a 10/66 population‐based cohort study. BMC Med. 2015;13(1):138. doi:10.1186/s12916-015-0378-4 26063168 PMC4481121

[20] Banerjee J , Jain U , Khobragade P , et al. Methodological considerations in designing and implementing the harmonized diagnostic assessment of dementia for longitudinal aging study in India (LASI‐DAD). Biodemography Soc Biol. 2020;65(3):189‐213. doi:10.1080/19485565.2020.1730156 32727279 PMC7398273

[21] Abellan van Kan G , Rolland Y , Bergman H , Morley JE , Kritchevsky SB , Vellas B . The I.A.N.A Task Force on frailty assessment of older people in clinical practice. J Nutr Health Aging. 2008;12(1):29‐37. doi:10.1007/BF02982161 18165842

[22] Mitnitski AB , Mogilner AJ , Rockwood K . Accumulation of deficits as a proxy measure of Aging. Sci World J. 2001;1:323‐336. doi:10.1100/tsw.2001.58 PMC6084020 12806071

[23] Searle SD , Mitnitski A , Gahbauer EA , Gill TM , Rockwood K . A standard procedure for creating a frailty index. BMC Geriatr. 2008;8(1):24. doi:10.1186/1471-2318-8-24 18826625 PMC2573877

[24] Song X , Mitnitski A , Rockwood K . Prevalence and 10‐year outcomes of frailty in older adults in relation to deficit accumulation. J Am Geriatr Soc. 2010;58(4):681‐687. doi:10.1111/j.1532-5415.2010.02764.x 20345864

[25] Wilde MH , McMahon JM , Crean HF , Brasch J . Exploring relationships of catheter associated urinary tract infection and blockage in people with long‐term indwelling urinary catheters. J Clin Nurs. 2017;26(17–18):2558‐2571. doi:10.1111/jocn.13626 27805758 PMC5413425

[26] Cheng FW , Gao X , Mitchell DC , et al. Body mass index and all‐cause mortality among older adults. Obesity. 2016;24(10):2232‐2239. doi:10.1002/oby.21612 27570944

[27] Biritwum RB , Minicuci N , Yawson AE , et al. Prevalence of and factors associated with frailty and disability in older adults from China, Ghana, India, Mexico, Russia and South Africa. Maturitas. 2016;91:8‐18. doi:10.1016/j.maturitas.2016.05.012 27451316

[28] Kshatri JS , Palo SK , Bhoi T , Barik SR , Pati S . Associations of multimorbidity on frailty and dependence among an elderly rural population: findings from the AHSETS study. Mech Ageing Dev. 2020;192:111384. doi:10.1016/j.mad.2020.111384 33080280

[29] Kendhapedi KK , Devasenapathy N . Prevalence and factors associated with frailty among community‐dwelling older people in rural Thanjavur district of South India: a cross‐sectional study. BMJ Open. 2019;9(10):e032904. doi:10.1136/bmjopen-2019-032904 PMC6797472 31604789

[30] Siriwardhana DD , Hardoon S , Rait G , Weerasinghe MC , Walters KR . Prevalence of frailty and prefrailty among community‐dwelling older adults in low‐income and middle‐income countries: a systematic review and meta‐analysis. BMJ Open. 2018;8(3):e018195. doi:10.1136/bmjopen-2017-018195 PMC5855322 29496895

[31] Ofori‐Asenso R , Chin KL , Mazidi M , et al. Global incidence of frailty and prefrailty among community‐dwelling older adults: a systematic review and meta‐analysis. JAMA Netw Open. 2019;2(8):e198398. doi:10.1001/jamanetworkopen.2019.8398 31373653 PMC6681553

[32] Nguyen T , Cumming RG , Hilmer SN . A review of frailty in developing countries. J Nutr Health Aging. 2015;19(9):941‐946. doi:10.1007/s12603-015-0503-2 26482697

[33] Hoogendijk EO , Rijnhart JJM , Kowal P , et al. Socioeconomic inequalities in frailty among older adults in six low‐ and middle‐income countries: results from the WHO Study on global AGEing and adult health (SAGE). Maturitas. 2018;115:56‐63. doi:10.1016/j.maturitas.2018.06.011 30049348

[34] Franse CB , van Grieken A , Qin L , Melis RJF , Rietjens JAC , Raat H . Socioeconomic inequalities in frailty and frailty components among community‐dwelling older citizens. PLoS One. 2017;12(11):e0187946. doi:10.1371/journal.pone.0187946 29121677 PMC5679620

[35] Coovadia H , Friedman I . Reducing health inequalities in developing countries. In: Detels R , Gulliford M , Karim QA , Tan CC , eds. Oxford Textbook of Global Public Health. 6th ed. Oxford University Press; 2015:127‐139. Accessed February 23, 2022. https://oxfordmedicine.com/view/10.1093/med/9780199661756.001.0001/med‐9780199661756‐chapter‐9

[36] Llibre Rodriguez JJ , Prina AM , Acosta D , et al. The prevalence and correlates of frailty in urban and rural populations in Latin America, China, and India: a 10/66 population‐based survey. J Am Med Dir Assoc. 2018;19(4):287‐295.e4. doi:10.1016/j.jamda.2017.09.026 29306607

[37] Pradhananga S , Regmi K , Razzaq N , Ettefaghian A , Dey AB , Hewson D . Ethnic differences in the prevalence of frailty in the United Kingdom assessed using the electronic frailty index. Aging Med. 2019;2(3):168‐173. doi:10.1002/agm2.12083 PMC6880682 31942531

[38] Gupta M , Brister S , Verma S . Is South Asian ethnicity an independent cardiovascular risk factor? Can J Cardiol. 2006;22(3):193‐197. doi:10.1016/s0828-282x(06)70895-9 16520847 PMC2528919

[39] Chaudhary M , Arokiasamy P . Patterns of frailty and quality of life among older adults: comparative analysis using SAGE states of India. J Popul Ageing. 2019;12(1):1‐23. doi:10.1007/s12062-017-9201-7

[40] Anand A , Syamala TS , Sk MK , Bhatt N . Understanding frailty, functional health and disability among older persons in India: a decomposition analysis of gender and place of resident. J Res Health Sci. 2020;20(3):e00484. doi:10.34172/jrhs.2020.20 33169716 PMC7585762

[41] Chaudhary M , Chowdhary R . Age and socioeconomic gradients in frailty among older adults in India. J Public Health. 2019;27(5):675‐685. doi:10.1007/s10389-018-0988-3

[42] Health performance: NITI Aayog, National Institution for Transforming India, Government of India. Accessed February 23, 2022. http://social.niti.gov.in/hlt‐ranking

[43] Global health estimates: leading causes of death. Accessed February 23, 2022. https://www.who.int/data/maternal‐newborn‐child‐adolescent‐ageing/advisory‐groups/gama/gama‐related‐resources/gho

[44] Chi J , Chen F , Zhang J , et al. Impacts of frailty on health care costs among community‐dwelling older adults: a meta‐analysis of cohort studies. Arch Gerontol Geriatr. 2021;94:104344. doi:10.1016/j.archger.2021.104344 33516075

[45] Bock JO , König HH , Brenner H , et al. Associations of frailty with health care costs – results of the ESTHER cohort study. BMC Health Serv Res. 2016;16:128. doi:10.1186/s12913-016-1360-3 27074800 PMC4831082

[46] Chang SF , Lin HC , Cheng CL . The relationship of frailty and hospitalization among older people: evidence from a meta‐analysis. J Nurs Scholarsh. 2018;50(4):383‐391. doi:10.1111/jnu.12397 29874399

[47] Kojima G . Frailty as a predictor of hospitalisation among community‐dwelling older people: a systematic review and meta‐analysis. J Epidemiol Community Health. 2016;70(7):722‐729. doi:10.1136/jech-2015-206978 26933121

[48] Kowal P , Afshar S . Health and the Indian caste system. Lancet. 2015;385(9966):415‐416. doi:10.1016/S0140-6736(15)60147-7 25706969

