# Supplementary material for: The prevalence of frailty and its relationship with sociodemographic factors, regional healthcare disparities, and healthcare utilization in the aging population across India
Source: Aging Med (Milton). 2023 Aug 7;6(3):212–21. doi: 10.1002/agm2.12263 (PMC10498834; doi:10.1002/agm2.12263)
Supplement: Supplementary file 1 — Appendix S1. [file AGM2-6-212-s001.docx]

**Supplementary Table S1. Items and scoring scheme for the frailty index**

|  | **Sub-section** | **Item** | **Response** | **Score** |
| --- | --- | --- | --- | --- |
|  | Diagnosed Health Conditions | Stroke | No | 0 |
|  |  |  | Yes | 1 |
|  |  | Parkinson | No | 0 |
|  |  |  | yes | 1 |
|  |  | Memory problems | No | 0 |
|  |  |  | Yes | 1 |
|  | BLESSED Test (Part 2) | Eating | Feeding self without assistance | 0 |
|  |  |  | Feeds self with assistance (include minor/much) | 0.5 |
|  |  |  | Has to be fed | 1 |
|  |  | Toilet | Clean, care for self | 0 |
|  |  |  | Incontinence (include occasional and frequent) | 0.5 |
|  |  |  | Little or no control | 1 |
|  |  | Dressing | Unaided | 0 |
|  |  |  | Some difficulty (occasional misplace buttons, wrong sequence) | 0.5 |
|  |  |  | Unable to dress | 1 |
|  | Everyday Activities | Able to travel | Yes | 0 |
|  |  |  | No | 1 |
|  |  | Daily activities | No change | 0 |
|  |  |  | Slowing down | 0.5 |
|  |  |  | Activities decrease or discontinue | 1 |
|  | Cognitive Activity Score (CSI) | General decline in mental functioning | No | 0 |
|  |  |  | Yes | 1 |
|  | Blood Pressure Measurements | High blood pressure (systolic mean >140 mmHg) | No | 0 |
|  |  |  | Yes | 1 |
|  | Anthropometry | Body mass index, Kg/m^2^ | 18.5 to <25 | 0 |
|  |  |  | 25 to <30 | 0.50 |
|  |  |  | <18.5 or ≥30 | 1 |
|  |  | Whether able to stand for measurements | Yes | 0 |
|  |  |  | No | 1 |
|  | Activities of daily living (ADLs): Some difficulty | Difficulty walking across room (ADL) | No difficulty | 0 |
|  |  |  | Any difficulty | 1 |
|  |  | Bathing | No difficulty | 0 |
|  |  |  | Any difficulty | 1 |
|  | Instrumental activities of daily living (IADLs): Some difficulty | Use of telephone | No difficulty | 0 |
|  |  |  | Any difficulty | 1 |
|  |  | Take medication | No difficulty | 0 |
|  |  |  | Any difficulty | 1 |
|  |  | Doing household chores | No difficulty | 0 |
|  |  |  | Any difficulty | 1 |
|  |  | Getting around | No difficulty | 0 |
|  |  |  | Any difficulty | 1 |
|  | Mental Health (CESD Score) | Felt depressed | Rarely or never | 0 |
|  |  |  | Sometimes/often | 0.5 |
|  |  |  | Most or all the time | 1 |
|  |  | Felt tired | Rarely or never | 0 |
|  |  |  | Sometimes/often | 0.5 |
|  |  |  | Most or all the time | 1 |
|  |  | Everything was an effort | Rarely or never | 0 |
|  |  |  | Sometimes/often | 0.5 |
|  |  |  | Most or all the time | 1 |
|  | Anxiety Inventory | Fear of dying (anxiety) | Never | 0 |
|  |  |  | Hardly/some of the time | 0.5 |
|  |  |  | Most of the time | 1 |
|  | Mini-Nutritional assessment (MNA) | Decrease in food intake last 3 months | No decrease | 0 |
|  |  |  | Moderate decrease | 0.5 |
|  |  |  | Severe decrease | 1 |
|  |  | Weight loss | No loss/ 1-3kg/does not know | 0 |
|  |  |  | >3kg | 1 |
|  |  | Mobility | Goes out | 0 |
|  |  |  | Able to get out of bed/chair but does not go out | 0.5 |
|  |  |  | Bed or chair bound | 1 |
|  |  | >3 prescription drugs | No | 0 |
|  |  |  | Yes | 1 |
|  |  | Pressure sore/skin ulcer | No | 0 |
|  |  |  | Yes | 1 |
|  |  | Self-viewed nutrition status | Views no nutritional/uncertain problem | 0 |
|  |  |  | Views self as being malnourished | 1 |
|  |  | Self-rated health status | Better/ As good / Does not know | 0 |
|  |  |  | Not as good | 1 |
|  |  | Mid-arm circumference | >22 cm | 0 |
|  |  |  | 0-22 cm | 1 |
|  |  | Calf circumference | >31 cm | 0 |
|  |  |  | 0-31 cm | 1 |
|  | Hearing Tests | Hearing test | Good (good acuity in both ears) or Mild difficulty (mild difficulty in both or either ear) | 0 |
|  |  |  | Moderate to severe difficulty  (moderate to severe difficulty in both or either ear) | 1 |

**Supplementary Table S2. The frailty index and its components in high-performing, intermediate-performing, and low-performing states**

|  | **High Performing states – n (%)** | **Intermediate Performing states – n (%)** | **Low Performing states – n (%)** | **p-value** |
| --- | --- | --- | --- | --- |
| Frailty Index (Mean ± SE)* | 20.9 ± 0.4 | 24.9 ± 0.4 | 26.0 ± 0.5 | <0.001 |
| Frail* | 558 (34.5) | 628 (46.8) | 583 (49.7) | <0.001 |
| Frailty Components | | | | |
| Stroke | 66 (4.39) | 94 (7.13) | 69 (6.17) | 0.007 |
| Parkinson | 27 (1.80) | 24 (1.83) | 89 (7.91) | <0.001 |
| Memory problems | 82 (5.46) | 197 (15.04) | 176 (15.74) | <0.001 |
| Eating, has to be fed | 7 (0.47) | 47 (3.56) | 2 (0.18) | <0.001 |
| Toilet, little or no control | 14 (0.93) | 8 (0.61) | 1 (0.09) | 0.001 |
| Dressing, unable to dress | 14 (0.93) | 12 (0.91) | 9 (0.80) | 0.002 |
| Able to travel | 510 (33.95) | 559 (42.32) | 441 (39.23) | <0.001 |
| Daily activities, activities decrease or discontinue | 156 (10.38) | 189 (14.36) | 180 (16.04) | <0.001 |
| General decline in mental functioning | 300 (20.00) | 390 (29.66) | 423 (37.90) | <0.001 |
| High blood pressure (systolic mean >140 mmHg) | 710 (47.65) | 597 (45.75) | 460 (41.97) | 0.016 |
| Body mass index, <18.5 or ≥30 Kg/m^2^ | 355 (25.56) | 408 (33.33) | 366 (34.76) | <0.001 |
| Not able to stand for measurements | 42 (2.82) | 43 (3.33) | 30 (2.69) | 0.608 |
| Difficulty Walking across room (ADL) | 306 (20.36) | 380 (28.77) | 328 (29.05) | <0.001 |
| Bathing | 203 (13.51) | 257 (19.45) | 208 (18.42) | <0.001 |
| Use of telephone | 562 (37.82) | 477 (37.50) | 613 (55.23) | <0.001 |
| Take medications | 188 (12.52) | 241 (18.24) | 229 (20.30) | <0.001 |
| Doing household chores | 476 (31.69) | 407 (30.93) | 432 (38.54) | <0.001 |
| Getting around | 581 (38.68) | 538 (41.01) | 540 (48.04) | <0.001 |
| Felt depressed, Most or all the time | 137 (9.13) | 90 (6.83) | 115 (10.29) | 0.008 |
| Felt tired, Most or all the time | 203 (13.51) | 160 (12.11) | 253 (22.51) | <0.001 |
| Everything was an effort, Most or all the time | 167 (11.17) | 117 (8.92) | 86 (7.73) | 0.009 |
| Fear of dying(anxiety), Most of the time | 24 (1.6) | 61 (4.63) | 35 (3.13) | <0.001 |
| Decrease in food intake last 3 months, Severe decrease | 172 (11.44) | 183 (13.87) | 124 (11.02) | <0.001 |
| Weight loss | 115 (7.68) | 134 (10.20) | 98 (8.74) | 0.063 |
| Mobility, bed or chair bound | 31 (2.06) | 66 (5.00) | 14 (1.24) | <0.001 |
| >3 prescription drugs | 363 (24.22) | 321 (24.32) | 128 (11.36) | <0.001 |
| Pressure sore/skin ulcer | 180 (11.98) | 88 (6.67) | 86 (7.64) | <0.001 |
| Self-viewed nutrition status | 234 (15.61) | 330 (25.10) | 317 (28.23) | <0.001 |
| Self-rated health status | 331 (22.05) | 377 (28.71) | 342 (30.48) | <0.001 |
| Mid-arm circumference ≤ 22 cm | 291 (19.57) | 304 (23.05) | 242 (21.49) | 0.079 |
| Calf circumference ≤ 31 cm | 868 (58.14) | 819 (62.09) | 834 (74.46) | <0.001 |
| Hearing | 675 (45.89) | 655 (51.86) | 502 (46.14) | 0.003 |

*Weighted values; SE- Standard Error

85 duplicate variables excluded

Final constructed LASI-FI:

32 health deficits

36 LASI-DAD variables considered for frailty index

260 LASI-DAD variables to be converted into health deficits

345 health-related variables

All LASI-DAD variables

(n = 392)

25 Demographic variables and 22 food-related variables excluded

260 LASI-DAD variables combined to create 36 health deficits

4 excluded due to missing data

**Supplementary Figure S1. Flow diagram describing the construction of frailty index.**


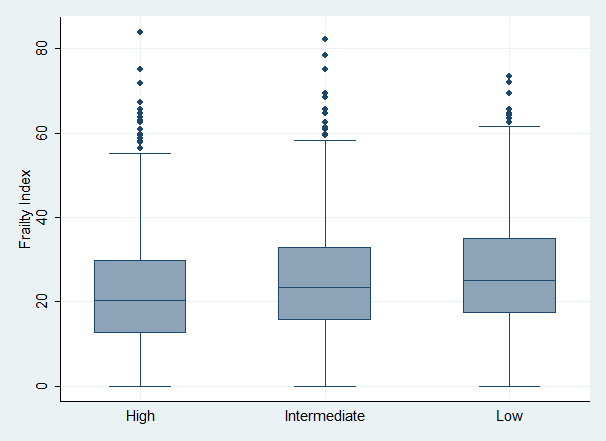


**Supplementary Figure S2. Distribution of frailty index in high-, intermediate-, and low-performing states.**
